# Efficacy of Autologous Bone Marrow Concentrate for Knee Osteoarthritis with and without Adipose Graft

**DOI:** 10.1155/2014/370621

**Published:** 2014-09-07

**Authors:** Christopher Centeno, John Pitts, Hasan Al-Sayegh, Michael Freeman

**Affiliations:** ^1^Regenerative Sciences, The Centeno-Schultz Clinic, 403 Summit Boulevard, Suite 201, Broomfield, CO 80021, USA; ^2^Forensic Research and Analysis, Oregon Health and Science University, 425 NW 10th Avenue, Suite 306, Portland, OR 97209, USA

## Abstract

*Introduction.* We investigated the use of autologous bone marrow concentrate (BMC) with and without an adipose graft, for treatment of knee osteoarthritis (OA). *Methods.* Treatment registry data for patients who underwent BMC procedures with and without an adipose graft were analyzed. Pre- and posttreatment outcomes of interest included the lower extremity functional scale (LEFS), the numerical pain scale (NPS), and a subjective percentage improvement rating. Multivariate analyses were performed to examine the effects of treatment type adjusting for potential confounding factors. The frequency and type of adverse events (AE) were also examined. *Results.* 840 procedures were performed, 616 without and 224 with adipose graft. The mean LEFS score increased by 7.9 and 9.8 in the two groups (out of 80), respectively, and the mean NPS score decreased from 4 to 2.6 and from 4.3 to 3 in the two groups, respectively. AE rates were 6% and 8.9% in the two groups, respectively. Although pre- and posttreatment improvements were statistically significant, the differences between the groups were not. *Conclusion.* BMC injections for knee OA showed encouraging outcomes and a low rate of AEs. Addition of an adipose graft to the BMC did not provide a detectible benefit over BMC alone.

## 1. Introduction

Knee osteoarthritis (OA) is a significant health problem with increasing impact on public health [1]. In 2009 there were approximately 600,000 total knee arthroplasties (TKAs) performed for knee OA, more than double the number performed 10 years earlier [2]. Total or partial joint arthroplasty surgeries are highly invasive procedures, requiring surgical resection of all or parts of the joint and insertion of a prosthesis [3]. Complications can be significant and include death, pulmonary embolism, stroke, and myocardial infarction [4–7]. While many patients who undergo TKA experience improved function and decreased symptoms, many others continue to have some degree of ongoing pain. A recent investigation of post-TKA symptoms reported chronic pain in 88% of patients who have had the surgery [8].

Nonsurgical alternatives to joint arthroplasty such as hyaluronic acid (HA) injections for knee OA are appealing due to lower cost and decreased morbidity [9, 10]. The treatment is less effective in patients with more severe disease and often only provides temporary relief lasting a few months [9, 10]. Autologous biologic therapies are also promising, with early data showing that platelet rich plasma (PRP) injection for knee OA may be of benefit for patients with mild to moderate osteoarthritis [11]. Two recent trials of HA versus PRP injections for knee OA demonstrated the superiority of PRP [12, 13]. However, PRP is less effective for patients with more severe OA [11].

Injection of autologous stem cells into the knee is a potentially promising treatment for moderate to severe OA. Mesenchymal stem cells are readily available in an outpatient setting and can be accessed via needle aspiration from a patient's bone marrow, as well as other sources [14]. Mesenchymal stem cells are multipotent and thus have the capability of differentiating into cartilage and bone [14, 15]. Early clinical studies using both isolated mesenchymal stem cells and bone marrow aspirate concentrate to treat osteoarthritis have been encouraging [16–19]. Another tissue that is a rich source of stem cells is adipose tissue [20, 21]. Several small studies have reported encouraging clinical results using adipose processed stromal vascular fraction (SVF) [22, 23]. Stem cell treatments could potentially provide a safe, less invasive, and nonsurgical treatment for knee OA; however, limited evidence for efficacy of this type of treatment exists in the literature. The purpose of this study is to expand the literature on safety and efficacy of BMC treatment of knee OA and explore whether adding adipose affects the results.

In the present study we evaluated the safety, efficacy, and differences of two stem cell therapies for knee OA using data gathered from a treatment registry. The first therapy was a same-day procedure using autologous bone marrow aspirate concentrate (BMC) alone, and the second was also a same-day procedure using BMC, but with the addition of adipose-derived lipoaspirate.

## 2. Methods

### 2.1. Setting and Participants

This is a longitudinal analysis of prospectively gathered registry data. We used a private knee registry, which is an ongoing prospective survey system that was designed to follow up specific protocols of autologous mesenchymal stem cells, bone marrow concentrate, and platelets rich plasma based treatment. Registry data for all patients who underwent a BMC procedure for knee OA from April 2010 to December 2013 were included in the study. Only patients who had responded to the outcome and complications questionnaires at 1 month and 3, 6, and 12 months following the procedure were included. There were 17 outpatient facilities that contributed patients to the registry, although the majority of cases (67.9%) were performed at a single center at which the primary author (CJC) is affiliated. Two patient groups were followed: the first received BMC and platelet rich plasma using a specified treatment protocol described in (group A) and the second received the same therapy plus the addition of an adipose fat graft (the lipoaspirate) (group B). The treatment protocols are described in detail in the “Procedure Descriptions” section. The indications for the second cohort were similar to those for the first, and the addition of the fat graft was at the discretion of the clinician. The data was collected prospectively and analyzed retrospectively.

### 2.2. Data Sources

Patients were enrolled in a treatment registry and tracked prospectively via an electronic database system using ClinCapture software (Clinovo Clinical Data Solutions, Sunnyvale, California; http://www.clinovo.com/clincapture). The program includes an automated emailing system to send patients clinical outcome questionnaires at a predetermined posttreatment frequency. In the present study we tracked patient response to treatment by (1) a subjective improvement rating scale from −100% worse to 100% improved; (2) the lower extremity functional (LEFS) questionnaire; and (3) the numeric pain scale (NPS) for symptom severity. Complications were monitored by e-mail or during clinic visit preoperatively and at 1 month, 3 months, and 6 months and annually after the procedure by a dedicated registry staff. Nonresponders were contacted by phone and/or e-mail. We have previously published a number of studies using data from the same registry [17, 24–28].

### 2.3. Outcomes of Interest

The outcomes of interest were patients' report of any serious or other adverse events, percentage of reported positive or negative change on a −100 to 100% subjective improvement rating scale, changes in activities of daily living as measured by the lower extremity functional scale (see Table 1), and pain measured by the numeric pain scale (NPS) at set time points following the procedure. Patients were asked to rate their average pain during that week at the area being treated. The response was restricted to 0–10 scale with no decimals allowed. Marking 0 indicated no pain and marking 10 indicated the worst possible pain. For the improvement rating scale, patients were asked the following question: “Compared to your condition prior to the procedure, what percent difference have you seen in your condition?” The response would range from −100% worse to 100% better with 0% indicating no change. No decimals were allowed in this scale as well. Efficacy was measured by the intragroup changes in LEFS and NPS.

### 2.4. Adjudication of Adverse Events

Patients were sent questionnaires to elicit adverse events at 1 month, 3 months, and 6 months and annually. These questionnaires included the following questions: “Did you experience any complications you believe may be due to the procedure (i.e. infection, illness, etc.)? If “Yes”, please explain” and “Have you been diagnosed with any new illness since the procedure? If “Yes”, please explain.” Any untoward or unfavorable medical occurrence that was reported was sent to the physician for adjudication. The treating physician or one of the authors (CJC) then determined through patient interview or chart review, based on the US Department of Health and Human Services guidelines [29], whether the condition was preexisting, unexpected, mild/moderate/severe, related to the therapeutic agent or procedure, or resolved/ongoing/fatal.

### 2.5. Covariates

In our analysis we included and accounted for a number of potentially confounding variables including age, gender, BMI, and severity of disease. Age was categorized into 3 groups: 50 years or younger (referent group), 51–60 years, and older than 60 years. BMI was categorized into below 25 (referent group), 25–29.9, and 30 or higher. The baseline severity of OA as well as candidacy for the procedure was graded following the Kellgren-Lawrence (KL) scale [30], in which KL1 was assigned a “Good” candidacy grade, KL2 was assigned a “Fair” grade, and KL3 and KL4 were assigned a “Poor” grade. These categories match the imaging-determined disease severity [31].

### 2.6. Statistical Analyses

Baseline characteristics were described using the mean and standard deviation for continuous variables and frequency and proportion for categorical variables. Baseline differences between the two groups were assessed using Student's *t*-test for continuous variables and chi square test or Fisher's exact test for categorical variables. For adverse events, we reported the frequency of events in each group per category. Intragroup changes in NPS and LEFS (differences in the pre- and posttreatment scores) were evaluated using the Wilcoxon signed-rank test, a nonparametric test for dependent samples. We examined the outcomes differences between the study groups using the Wilcoxon rank-sum test, a nonparametric test for independent samples. Multivariate analyses were performed to examine the effects of treatment type adjusting for potential confounding factors (covariates) utilizing the logistic regression analysis of binary outcomes. Logistic regression modelling is a well-known statistical method commonly used in medical research for predicting dichotomous outcomes (such as improvement versus nonimprovement) and finding associations between independent and dependent variables [32, 33]. In this study, logistic regression was modeled for functional and symptomatic improvement, which was defined as a ≥9 point increase in the LEFS score, ≥2 points drop in the NPS score, and ≥50% improvement as measured on the improvement rating scale. The cut points for LEFS and NPS changes were selected based on the minimally important clinical differences (MICD) reported in the medical literature, while the 50% improvement cut point was selected arbitrarily [34, 35]. As no decimals were allowed in the NPS scale, the MICD was considered to be 2 instead of 1.2. The effects of treatment type and covariates were described by reporting the odds ratios and their 95% confidence intervals. Separate models were constructed for each dependent variable (LEFS, NPS, and improvement rating scale). The baseline scales were stratified into upper and lower groups to control for confounding due to baseline score differences. We were also interested to see whether different functional and symptomatic presenting groups demonstrate different effects and associations. Each group was then analyzed independently. We used the complete case analysis approach where subjects with missing observations were deleted. All analyses were performed utilizing the SAS 9.4 software [36].

### 2.7. Procedure Descriptions

#### 2.7.1. Preinjection for All Patients

The first step of the treatment was a preinjection of a hypertonic dextrose solution into the knee joint or other painful extra-articular knee structures two to five days prior to injection of the bone marrow concentrate. The purpose of this preinjection was to introduce a chemical irritant to the joint in order to prompt a brief inflammatory response. Intra-articular needle placement was confirmed on ultrasound or fluoroscopy. If fluoroscopy was used, Iodixanol (Visipaque, NDC# 0407-2223-06) radiographic contrast was injected to confirm intra-articular flow in the joint. This was followed by injection of 3–5 ccs of 12.5% dextrose (NDC# 0409-6648-02) and 0.1% lidocaine (NDC# 0409-4276-02) in normal saline (NDC# 0409-4888-50).

#### 2.7.2. Harvest and Preparation of the Bone Marrow Concentrate (Groups A and B)

All patients underwent a bone marrow aspiration. Prior to the procedure the patients were restricted from taking corticosteroids and nonsteroidal anti-inflammatory drugs (NSAIDs) for at least 2 weeks as these medications can reduce healing [37–41]. Whole bone marrow aspirate was harvested from the patients' posterior superior iliac crest under ultrasound or fluoroscopic guidance. Approximately 10–15 cc of bone marrow aspirate was withdrawn from 6–8 sites (approximately 3-4 on each side) into heparinized syringes. There were 1,000 units of heparin (NDC# 25021-403-01 and 25021-404-01) per 1 cc of whole bone marrow aspirate drawn into syringe. The aspirate was processed by hand in a sterile ISO-7 class clean room and in ISO-5 class laminar flow cabinets to isolate the buffy coat through centrifugation. This isolation produced 1–3 cc of BMC injectate which was then transported via sterile means back to the operating room. Coincident with this harvest procedure approximately 60 ccs of heparinized IV venous blood was drawn to be used for isolating platelet rich plasma (PRP) and platelet lysate (PL). To prepare the PRP, plasma was prepped via centrifugation at 200 g to separate plasma and buffy coat layers from the red blood cells. The resultant liquid lying above the concentrated solids (supernatant) was red cell/white cell poor. To prepare the PL, PRP was drawn off and stored at −20° Celsius; platelet bodies were recentrifuged, and the supernatant was drawn off.

#### 2.7.3. Reinjection of the Bone Marrow Concentrate (Groups A and B)

Needle placement into the joint was accomplished utilizing fluoroscopy or ultrasound as described above with intra-articular placement confirmed. The injectate consisted of bone marrow concentrate, PRP, and PL. This was injected intra-articularly and into painful or otherwise damaged structures. For example, if a meniscus tear was detected on MRI, the patient's meniscus was also injected under ultrasound guidance into areas of decreased echogenicity. Based on medical need, infrequent additional platelet rich plasma injections may have been provided by the treating physician.

After the procedure the patients were given activity instructions and bracing if they had one compartment dominant disease. A posttreatment off-loader brace was commonly prescribed for the most involved compartment with the patient being given instructions to wear the brace with all weight bearing activity for 6 weeks. For patella-femoral compartment dominant OA patients, a patellar stabilizer brace was used. Patients were discharged with instructions to be light weight bearing for several days if there was significant post-op pain but then to return to full weight bearing as soon as feasible. Post-op activity sheets were provided to the patient, which described a gradual return to full activities over 6 weeks. The patients were encouraged to participate in physical therapy, but this was not required nor controlled.

#### 2.7.4. Lipoaspirate Harvest and Reinjection (Group B Only)

For this subgroup, at the time of the bone marrow draw, a miniliposuction was performed under ultrasound guidance and minimally processed adipose tissue was injected into the articular space. For the harvest of adipose tissue, patients were placed prone or in the lateral decubitus position and an area in the posterior superior buttocks or lateral thigh was anesthetized. The area was imaged under ultrasound and a Tulip Twin Port Harvester (#harvtwn) was moved back and forth in the subcutaneous tissue to break up the adipose tissue. Approximately 5–15 cc of lipoaspirate was then drawn into a 60 cc syringe containing heparin (NDC# 25021-403-01 and 25021-402-01) and using a Tulip Snaplok (#Snap_60) to maintain suction. The lipoaspirate was minimally processed via low speed centrifugation or by allowing the layers to settle over several hours and the top oil layer was drawn off. The tissue was then injected into the articular space with the BMC using an 18 gauge needle between the meniscus on the most painful side and the over lying collateral ligament, at a volume of 5–10 cc.

## 3. Results

There were 616 procedures performed on 518 patients in group A and 224 procedures performed on 163 patients in group B, for a total of 840 treated knees in 681 patients. There were 98 and 61 patients who underwent bilateral procedures in groups A and B, respectively. Mean age, BMI, and percent male were 54.3 years, 26.5, and 64.5% in group A and 59.9, 27.0, and 53.1% in group B, respectively (Table 2). There were 196 (31.8%) bilateral procedures in group A and 122 (54.5%) bilateral procedures in group B. An additional 77 (12.5%) patients in group A and 25 (11.2%) patients in group B underwent additional PRP injections after their initial procedure. Radiographic data sufficient for OA severity classification were available for 646 out of 840 knees in both groups (76.9%). The majority of patients were Caucasian (White), constituting 89.3% of group A and 88.8% of group B.

Survey response rates for improvement rating scale were 66.2% (408 of 616 procedures) for group A and 74.1% (166 of 224 procedures) for group B. The averaged postprocedure time of last available reported improvement was 10.4 months for group A and 10.7 months for group B (Table 3). Mean reported improvement was 46.8% for group A and 39.3% for group B at final follow-up. The response rate for the last available LEFS was 33.3% at an average of 6.2 months for group A after procedure and 40.6% at 5.7 months for group B. The mean LEFS changes from baseline were 7.93 points for group A and 9.8 points for group B (*P* ≤ 0.001 for intragroup differences). The response rate for the NPS questionnaire was 35.7% at an average of 7.0 months for group A and 46.0% at 6.7 months for group B. Patients in group A reported a mean baseline pain level of 4.0/10 versus 2.6/10 at final follow-up. The 1.4 decrease in NPS scores is a 35.3% drop relative to baseline. Patients in group B reported a mean baseline of 4.3/10 versus 3.0/10 at final follow-up (*P* ≤ 0.001 for intragroup differences). The 1.31-point decrease is a 30.2% drop relative to baseline. The only intergroup difference in treatment response that reached statistical significance was the subjective percentage improvement scale.

Two additional subgroup analyses were performed in order to examine the differences among the patients undergoing bilateral procedures versus unilateral procedures, as well as those who received additional PRP treatment versus those who did not. Neither of the subgroups demonstrated significantly different results.

### 3.1. Survey Response Safety

Out of 840 procedures followed for an average of 17.7 months (range 1–41 months) there were 57 reported adverse events (AEs), including 37 in group A and 20 in group B (6% and 8.9% of total patients, resp.). AEs were categorized, frequency and outcomes recorded, and they were adjudicated to a relationship to the procedure (see Table 4 for details). No clear trends could be ascertained with regard to types of complaints and the different procedures. Three AEs (rate of 0.4% of all patients) were graded as severe; however, none were adjudicated to be secondary to the procedure, nor were they associated with reports of ongoing disability.

### 3.2. Logistic Regression Modeling

There were no statistically significant differences in outcomes between group A (reference group) and group B (Table 5). For both groups combined, outcome was significantly impacted by baseline LEFS score ≤45, gender, and BMI. Females were more likely to report improvement on the dichotomized LEFS scale compared to males [OR = 3.44 (1.5–8.2)] and dichotomized NPS outcomes [OR = 2.6 (1.0–6.6)]. The 2 higher BMI groups were more likely to report improvement on the LEFS scale as well, with an OR of 3.5 (1.2–9.8) for the 25–29.9 BMI group relative to the lowest BMI group and an OR of 3.0 (1.0–8.6) for the ≥30 BMI group relative to the lowest BMI group. Female patients were more likely to report a drop in the NPS score of 2 or more, an effect only detected among patients with baseline score of 5 or higher.

KL2 patients were significantly more likely (2.2 times) to report ≥50% improvement on the reported outcome scale in comparison with the reference group (KL3-4 grade).

## 4. Discussion

Self-rated functional and pain scores all showed statistically significant positive changes from baseline in both treatment groups. Although the LEFS score mean changes in both groups were greater than the potential error for the questionnaire of 5.3 points, only the mean change in group B was greater than the minimal clinically important difference (MCID) of 9 [34]. The mean change in group A did not exceed the MCID. The mean pain decrease of both knee OA groups exceeded the MCID for visual analog pain scales when applied to a Numerical Pain Scale (1–10 metric) of 1.2 points [35]. We were surprised to see that there was no obvious benefit from the addition of the lipoaspirate. For example, while group B met the MCID for the LEFS questionnaire, it showed a lesser drop in pain as measured by NPS. Both groups reported positive percent improvement, but group A patients reported a greater improvement rating than group B.

There was no correlation observed between age and outcome in the models, a finding that is in keeping with the variability seen in prior reports that have examined age related effects. As an example, in one study younger patients demonstrated better outcomes with knee microfracture procedure to treat osteochondral defects [42], and in another study autologous chondrocyte implantation was noted to demonstrate similar age related effects [43]. Other authors have found no such relationship for cartilage repair procedures [44]. There was also a correlation between lower severity of arthritis and better improvement on the subjective percentage improvement rating scale, but this correlation did not extend to the NPS or LEFS outcomes. Thus, we cannot easily interpret if severity of arthritis based on the KL grading scale is predictive of outcomes in this study.

In this present study, lower functioning female patients and those with higher pain levels were more likely to report an improvement in comparison with male patients. These findings are in keeping with results previously reported in treatment registry studies for total knee arthroplasty [45, 46] and also consistent with the previous reports indicating that women are less likely to need knee arthroplasty revision [47]. These findings are reported with caution, as significant differences were seen only in the LEFS and NPS metrics and not in the self-reported improvement scale. Thus, the real impact of the observed differences is difficult to characterize.

An unexpected finding was that higher BMI patients in the lower functioning subset were more likely to report functional improvement than lower BMI patients. The result is inconsistent with what prior authors have reported. For example, in a 2013 review, the authors reported poorer objective and functional scores in morbidly obese versus normal to obese patients following knee arthroplasty [48]. Obese patients are also known to experience more perioperative complications and increased failure rates with knee arthroplasty [49–51]. The relationship has not been reported consistently, however, as there are also reports in the literature for microfracture and total knee arthroplasty procedures that indicate that obesity is not a factor in outcomes [51, 52]. There is not a readily apparent explanation for our observation that obesity was a positive factor for functional outcomes.

Pain/swelling was the most commonly reported adverse event. This was generally self-limited and resolved without any intervention. There was no significant difference between group A and group B. There was a trend for more reported pain/swelling in group B, which could have been associated with the proinflammatory effects of adipose oil being placed into the joint [53] or it may have been artifactual. Seven patients reported ongoing pain/soreness complaints, which were deemed to be related to either the OA disease process or the treatment failure. There were no significant differences between the groups for such complaints.

The miscellaneous category of AEs included complaints such as clicking, popping, catching, or instability in the joint, a self-limited feeling of asymmetry, muscle cramping, and no improvement. There was minimal difference between the groups in this category. The skin category included self-limited itching/rash (1). Both patients who reported bleeding/hematoma had self-limited hematomas at the bone marrow aspirate site. One patient visited the emergency room and was imaged there, but treatment was with supportive care only. One patient in the immune/allergic category reported an acute unrelated viral infection with lethargy and another reported a self-limited allergic reaction to the skin anesthetic. There were two patients who reported neoplasm, but neither were in the lower extremity (breast and gastric).

The overall safety of the procedure was substantially better than for total knee arthroplasty. The serious adverse event rate in one registry study tracking >15,000 arthroplasty patients was reported at 5.6%, with a 0.2% mortality rate [54]. Even if all SAEs reported in our study were due to the procedures, the rate of 0.4% would be far less than the SAE rates reported for joint arthroplasty. In addition, even if the one mortality due to gastric cancer was adjudicated as being caused by the procedure, the mortality rate of 0.1% would be less than that commonly seen in arthroplasty procedures.

The results from the procedures described are limited by the fact that the data collection was via a prospective treatment registry. Thus, the analyses described here are based on self-reported data, which limits the internal validity of the study because of the increased chance of reporting bias [55]. The most significant limitation of the study is, of course, the lack of randomization of patients into a placebo or alternate treatment control group. Additionally, there was no randomization of the lipoaspirate treatment group versus the nonlipoaspirate group.

## 5. Conclusions

This report of registry data on two groups of patients receiving BMC injections for knee OA shows encouraging results. We found that the addition of a lipoaspirate to the bone marrow concentrate did not provide any measured benefit over BMC alone. The reported complications for the two therapy groups were very low and far less than those commonly reported for knee arthroplasty procedures. While our results are encouraging, more study is needed using randomized controlled trails to confirm the reported effects.

## Figures and Tables

**Table 1:** Lower extremity function scale (LEFS).

Instructions						
We are interested in knowing whether you are having any difficulty at all with the activities listed below because of your lower limb problem for which you are currently seeking attention. Please provide an answer for each activity.						
Today, *do you or would you* have any difficulty at all with the following:						
	Activities	Extremely difficult or unable to perform activity	Quite a bit of difficulty	Moderate difficulty	A little bit of difficulty	No difficulty

(1)	Any of your usual work, housework, or school activities	⬜0	⬜1	⬜2	⬜3	⬜4
(2)	Your usual hobbies, recreational or sporting activities	⬜0	⬜1	⬜2	⬜3	⬜4
(3)	Getting into or out of the bath	⬜0	⬜1	⬜2	⬜3	⬜4
(4)	Walking between rooms	⬜0	⬜1	⬜2	⬜3	⬜4
(5)	Putting on your shoes or socks	⬜0	⬜1	⬜2	⬜3	⬜4
(6)	Squatting	⬜0	⬜1	⬜2	⬜3	⬜4
(7)	Lifting an object, like a bag of groceries, from the floor	⬜0	⬜1	⬜2	⬜3	⬜4
(8)	Performing light activities around your home	⬜ 0	⬜1	⬜2	⬜3	⬜4
(9)	Performing heavy activities around your home	⬜0	⬜1	⬜2	⬜3	⬜4
(10)	Getting into or out of a car	⬜0	⬜1	⬜2	⬜3	⬜4
(11)	Walking 2 blocks	⬜0	⬜1	⬜2	⬜3	⬜4
(12)	Walking a mile	⬜0	⬜1	⬜2	⬜3	⬜4
(13)	Going up or down 10 stairs (about 1 flight of stairs)	⬜0	⬜1	⬜2	⬜3	⬜4
(14)	Standing for 1 hour	⬜0	⬜1	⬜2	⬜3	⬜4
(15)	Sitting for 1 hour	⬜0	⬜1	⬜2	⬜3	⬜4
(16)	Running on even ground	⬜0	⬜1	⬜2	⬜3	⬜4
(17)	Running on uneven ground	⬜0	⬜1	⬜2	⬜3	⬜4
(18)	Making sharp turns while running fast	⬜0	⬜1	⬜2	⬜3	⬜4
(19)	Hopping	⬜0	⬜1	⬜2	⬜3	⬜4
(20)	Rolling over in bed	⬜0	⬜1	⬜2	⬜3	⬜4

**Table 2:** Baseline characteristics of the study groups (group A: bone marrow concentrate (BMC), group B: BMC and adipose graft, BMI: body mass index, LEFS: lower extremity functional scale, NPS: numeric pain scale, SD: standard deviation, KL: Kellgren-Lawrence scale, and ∗indicates a statistically significant difference between groups).

	Group A		Group B		*P* value
	*N*	Mean (SD)	*N*	Mean (SD)	
Age	615	54.3 (14.1)	223	59.9 (10.3)	<0.001∗
BMI	561	26.5 (4.4)	202	27 (4.2)	0.039∗
Baseline LEFS	335	45.3 (15.9)	119	42.8 (14.5)	0.134
Baseline NPS	370	4.2 (2.4)	141	4.4 (2.4)	0.350

	Group A		Group B		*P* value
	*N*	%	*N*	%	

Gender	616		224		0.003∗
Male	397	(64.5)	119	(53.1)	
Female	219	(35.5)	105	(46.9)	
Grade	470		166		0.294
KL1	223	(48.5)	69	(41.6)	
KL2	145	(30.2)	58	(34.9)	
KL3-4	102	(21.3)	39	(23.5)	
Procedures per patient	616		224		<0.001∗
1 (unilateral)	420	(68.2)	102	(45.5)	
2 (bilateral)	196	(31.8)	122	(54.5)	

**Table 3:** Baseline, follow-up, and changes in the symptomatic and functional scales for patients with available baseline and follow-up data (group A: bone marrow concentrate (BMC), group B: BMC and adipose graft, LEFS: lower extremity functional scale, NPS: numeric pain scale, SD: standard deviation, and ∗indicates a statistically significant difference between groups).

	Group A		Group B		*P* value
	*N*	Mean (SD)	*N*	Mean (SD)	
Clinical scales					
Improvement rating scale	408	46.8 (38)	166	39.3 (39.8)	0.030∗
LEFS (baseline)	205	46.1 (15.8)	91	43.6 (14.9)	—
LEFS (follow-up)	205	54 (17.9)	91	53.4 (14.7)	—
NPS (baseline)	220	4 (2.3)	103	4.3 (2.0)	—
NPS (follow-up)	220	2.6 (2.3)	103	3 (2.3)	—
Changes from the baseline					
Change in LEFS	205	7.9 (16.1)	91	9.8 (14.2)	0.335
Change in NPS	220	−1.4 (2.6)	103	−1.3 (2.5)	0.761
Follow-up (in months)					
Improvement rating scale	408	10.4 (9.4)	166	10.7 (8.1)	—
LEFS	205	6.2 (5.0)	91	5.7 (3.8)	—
NPS	220	7 (6.6)	103	6.7 (5.3)	—

**Table 4:** Group A: bone marrow concentrate (BMC), Group B: BMC and adipose graft. Number of adverse events reported in each group classified by category, severity, relation to preexisting condition, procedure and injected component, and outcomes.

	Group A	Group B
Category		
Pain/swelling	23	13
Miscellaneous	7	2
Skin reactions	1	0
Neurologic	0	2
Neoplasm	2	0
Immune/allergic	2	0
Cardiac	0	2
Bleeding/hematoma	2	0
Renal	0	1
Severity		
Mild	26	14
Moderate	9	5
Severe	2	1
Related to preexisting condition	9	5
Relation to procedure		
Definitely related	4	5
Likely related	0	0
Possibly related	17	12
Unlikely related	11	2
Not related	5	1
Relation to injected components		
Definitely related	1	3
Likely related	0	0
Possible related	16	8
Unlikely related	14	4
Not related	6	5
Outcome		
Resolved/recovered	22	17
Ongoing	8	3
Not recovered	1	0
Fatal	2	0
Unknown	3	0
Not categorized	2	0
Total	**37 (6%)**	**20 (8.9%)**

**Table 5:** Adjusted odds ratios and 95% confidence intervals for lower extremity functional scale improvement (ΔLEFS ≥ 9 points), decreased NPS score (VAS drop ≥ 2 points), and reporting ≥50% improvement on the improvement rating scale (group A: bone marrow concentrate (BMC), group B: BMC and adipose graft, BMI: body mass index, LEFS: lower extremity functional scale, NPS: numeric pain scale, KL: Kellgren-Lawrence scale, ∗indicates a statistically significant odds ratio, and Ref: referent group).

	LEFS outcomes		NPS outcomes		Improvement rating scale outcomes
	Baseline LEFS ≤ 45 (*N* = 111)	Baseline LEFS = 46–70 (*N* = 102)	Baseline NPS ≥ 5 (*N* = 101)	Baseline NPS = 2–4 (*N* = 77)	(*N* = 422)
Group B	0.8 (0.3–2.1)	1.3 (0.5–3.6)	1.3 (0.5–3.2)	1.6 (0.6–4.6)	0.7 (0.4–1.1)
Group A	1 (Ref)	1 (Ref)	1 (Ref)	1 (Ref)	1 (Ref)
Age > 60	0.4 (0.2–1.3)	1.1 (0.4–2.9)	0.4 (0.1–1.3)	2.2 (0.6–8.5)	1.4 (0.9–2.3)
Age 51–60	0.4 (0.1–1.2)	0.6 (0.2–2)	0.3 (0.1–1.2)	2.5 (0.6–10.5)	1.5 (0.9–2.4)
Age ≤ 50	1 (Ref)	1 (Ref)	1 (Ref)	1 (Ref)	1 (Ref)
BMI ≥ 30	**3 (1.0**–**8.6)**∗	0.7 (0.1–4.1)	0.9 (0.3–3)	0.3 (0.1–1.2)	1 (0.6–1.8)
BMI 25–29.9	**3.5 (1.2**–**9.8 )**∗	1.7 (0.7–4.2)	1 (0.3–3.2)	0.4 (0.1–1.4)	1.2 (0.8–1.9)
BMI < 25	1 (Ref)	1 (Ref)	1 (Ref)	1 (Ref)	1 (Ref)
Female	**3.4 (1.5-8.2)** ∗	1.3 (0.6–3.2)	**2.6 (1.0**–**6.6)**∗	1.1 (0.4–2.9)	1.4 (0.9–2.1)
Male	1 (Ref)	1 (Ref)	1 (Ref)	1 (Ref)	1 (Ref)
KL1	0.9 (0.3–3)	1.6 (0.4–6.1)	1 (0.3–3.2)	0.9 (0.2–4.6)	1.7 (0.9–2.9)
KL2	0.9 (0.3–2.7)	2.2 (0.6–8.6)	1 (0.3–3.2)	0.8 (0.1–4.2)	**2.2 (1.2**–**3.9)**∗
KL3-4	1 (Ref)	1 (Ref)	1 (Ref)	1 (Ref)	1 (Ref)
